# The Successful Anesthetic Management of an Adult With Cri-du-Chat Syndrome by Using Personalized Behavioral Strategies: A Case Report

**DOI:** 10.7759/cureus.91969

**Published:** 2025-09-10

**Authors:** Paraskevi Mavridou, Christos Exarchos, Pinelopi Kitsakou, Panagiota Panagiotou

**Affiliations:** 1 Department of Anesthesiology, General Hospital of Ioannina "G. Hatzikosta", Ioannina, GRC

**Keywords:** airway management, behavior therapy, cri-du-chat syndrome, dental care, general anesthesia, intellectual disability

## Abstract

Cri-du-Chat syndrome (CDCS) is a rare genetic disorder caused by a partial deletion of the short arm of chromosome 5, and it is characterized by craniofacial dysmorphism, severe intellectual disability, and behavioral challenges. Anesthetic management in adults with CDCS is rarely reported, as the literature mostly focuses on pediatric cases. We describe the case of a 34-year-old male with CDCS who was scheduled for periodontal surgery under general anesthesia; he refused all pharmacological premedication and exhibited severe separation anxiety. A caregiver-guided behavioral approach using repetitive familiar phrases enabled a calm transfer to the operating room and successful intravenous access. Anticipating a difficult airway due to micrognathia, macroglossia, and a high Mallampati score (III), nasotracheal intubation was achieved uneventfully. Anesthesia was maintained with sevoflurane and opioid-free analgesia. Recovery was smooth, and same-day discharge was accomplished. This report emphasizes the importance of non-pharmacological preparation, meticulous airway planning, and fast-track anesthetic strategies in adult CDCS patients who cannot tolerate conventional premedication.

## Introduction

Cri-du-Chat syndrome (CDCS), first described by Lejeune et al. in 1963 [1], is caused by a partial deletion of chromosome 5p and has an estimated incidence of 1 in 15,000 to 1 in 50,000 live births [2]. Typical features of CDCS include a high-pitched “cat-like” cry, craniofacial anomalies (micrognathia, hypertelorism), hypotonia, developmental delay, and intellectual disability [2,3]. These features, along with neuromotor dysfunction and severe behavioral and cognitive impairments, increase the risk of difficult airway management, limit patient cooperation, and may necessitate alternative strategies when premedication is refused or poorly tolerated [4-7]. There is scarce anesthetic-related literature on adult CDCS patients undergoing general anesthesia, with most reports focusing on children and only a few describing adults [6,8]. We report the successful anesthetic management of an adult patient with CDCS and profound intellectual disability undergoing periodontal surgery under general anesthesia, without pharmacological premedication, as the patient preoperatively refused all pharmacological interventions, by using a uniquely individualized behavioral approach.

Written informed consent was obtained from the patient’s legal guardians for publication of this case report, including clinical details and images. A signed copy of the consent form has been submitted to the Cureus Editorial Office and is available upon request.

## Case presentation

A 34-year-old male, weighing 65 kg, with CDCS was scheduled for extensive periodontal treatment under general anesthesia. He had been born prematurely at 35 weeks and exhibited characteristic features at birth, including distinct facial features, micrognathia, hypertelorism, a high-pitched cry, and a single palmar crease. Due to these signs and his prematurity, he had undergone evaluation at three months of age, and the diagnosis of CDCS (46,XY,5p-) had been confirmed by peripheral blood lymphocyte karyotype analysis. He had no family history of genetic disorders, and both parents’ karyotypes were normal. His past medical history included hypothyroidism managed with levothyroxine, abnormal gait secondary to neuromotor dysfunction, and no prior exposure to anesthesia or related complications. There were no known drug allergies, seizures, or cardiac disease. Previous dental procedures had been limited to non-invasive care, resulting in poor oral hygiene that necessitated the planned extensive periodontal intervention under general anesthesia with nasotracheal intubation.

The patient’s functional status was significantly limited by profound intellectual disability. Communication was restricted to a few single words, and he relied heavily on his parents for daily care. The parents reported heightened anxiety in unfamiliar environments, resistance to touch by strangers, and previous episodes of aggression when separated from them.

**Figure 1:**
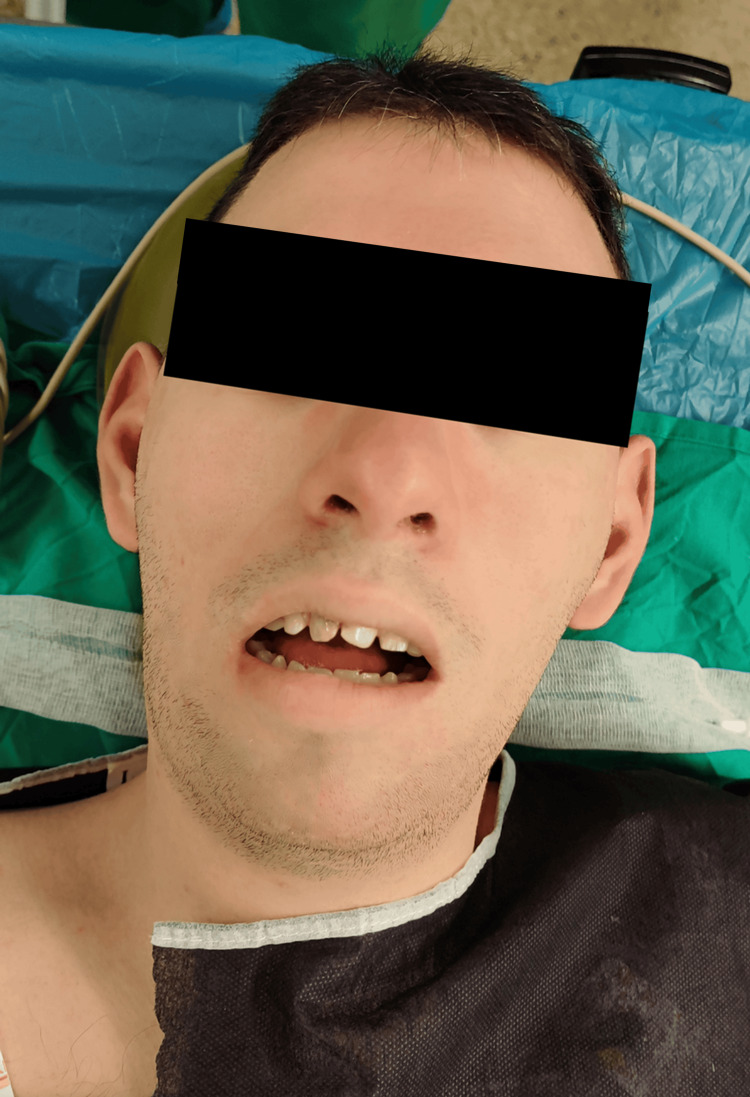
Characteristic craniofacial features of the patient with cri-du-chat syndrome

On admission, laboratory tests obtained in an external laboratory under parental supervision showed normal results, including thyroid function. Preoperative electrocardiography demonstrated sinus rhythm at 85 bpm. Airway examination was performed visually only, due to the patient’s refusal of detailed evaluation, and revealed Mallampati III, micrognathia, short thyromental distance, protruding incisors, and macroglossia (Figure 1), indicating potential difficulty with laryngoscopy and intubation. Mouth opening was adequate at 3 cm, and neck mobility was full.

Transfer to the operating room posed the greatest challenge, as the patient refused oral or intramuscular sedatives, and inhalational induction was impractical due to his tendency to push masks away forcefully and become aggressive. He exhibited severe separation anxiety and escalating agitation upon attempted removal from his parents’ side. Following a detailed meeting with caregivers, it was noted that specific stereotyped phrases and a simple rhythmic children’s song consistently calmed him. The anesthetic team incorporated these cues into the perioperative plan. On the day of surgery, the anesthesiologist and theater nurse repeatedly used these familiar phrases while holding the patient’s hands, allowing him to walk calmly into the operating room, accompanied by one parent up to the entrance. Continuous repetition of the song and phrases facilitated smooth cooperation while the patient remained seated for the application of standard monitors and successful intravenous cannulation on the first attempt by an experienced anesthesiologist, enabling immediate administration of induction agents. Monitoring included electrocardiography, pulse oximetry, non-invasive blood pressure measurement, temperature monitoring, and train-of-four (TOF) monitoring.

**Figure 2:**
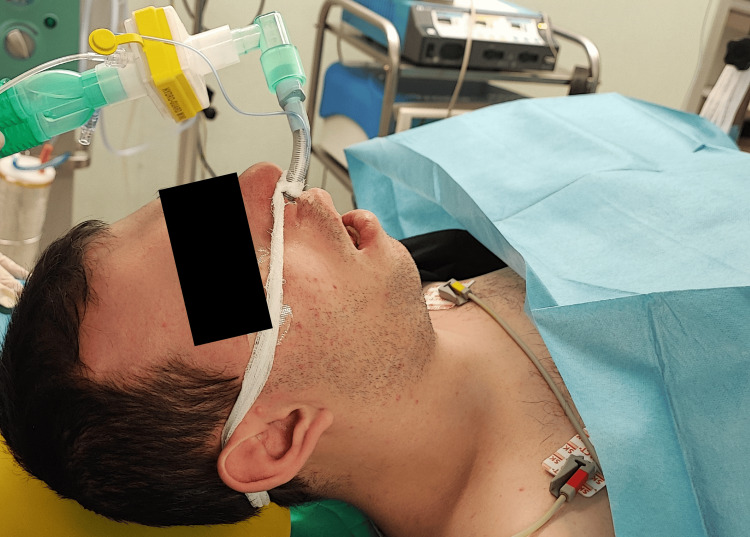
The patient immediately after successful nasotracheal intubation

Anesthesia was induced with propofol 2.5 mg/kg and rocuronium 1 mg/kg after preoxygenation. Given the potential difficulty with intubation, a difficult-airway cart was prepared, including a video laryngoscope, fiberoptic bronchoscope, various supraglottic devices, and surgical airway equipment. The more patent nostril was selected for nasotracheal intubation, performed with a lubricated 7.0 mm cuffed tube using Macintosh laryngoscopy and Magill forceps, achieving a Cormack-Lehane grade II view. No adjuncts were required, and nasotracheal intubation was completed without complications (Figure 2).

Maintenance of anesthesia was achieved with sevoflurane at an end-tidal concentration of 1.2 MAC in a 50% air-oxygen mixture, with intermittent boluses of rocuronium guided by TOF monitoring. Dexamethasone 8 mg was administered for prophylaxis of airway edema, and ondansetron 4 mg for prevention of postoperative nausea and vomiting. Intraoperative analgesia was provided with intravenous paracetamol 1 g and intermittent local infiltration with articaine by the dental team throughout the procedure. Hemodynamic parameters remained stable throughout the 215-minute surgery, with no episodes of desaturation or hypercapnia observed; in addition, temperature was continuously monitored via an esophageal probe, while warming blankets and fluid warmers were used to maintain normothermia.

At the end of the procedure, neuromuscular blockade was reversed with sugammadex 2 mg/kg, resulting in rapid restoration of muscle strength. The patient was extubated uneventfully when fully awake. In the post-anesthesia care unit, mild restlessness resolved quickly upon reunion with both parents. He was discharged home the same day, approximately seven hours after surgery, meeting all criteria for safe outpatient release.

## Discussion

CDCS is a rare genetic disorder characterized by distinctive craniofacial dysmorphism, severe intellectual disability, and varying degrees of developmental and behavioral abnormalities [3,4]. These features make the administration of general anesthesia particularly challenging. Furthermore, the rarity of the syndrome is reflected in the limited clinical experience and scarce literature, especially concerning anesthetic management in adult patients. Most published cases involve children, making adult management less standardized [8]. The anesthetic management of CDCS patients, particularly adults, demands comprehensive planning to address airway, behavioral, and recovery challenges. A significant issue is the presence of craniofacial anomalies that predispose to difficult intubation [4,8]. It is noteworthy that approximately 30% of surgical procedures in patients with CDCS involve dental interventions, primarily due to difficulties in maintaining oral hygiene [6]. Because of poor cooperation, these patients are typically managed as outpatients under general anesthesia, a practice that necessitates rapid recovery and timely hospital discharge [9].

The frequent need for nasotracheal intubation in dental procedures (to provide a better surgical field and protect the endotracheal tube during the procedure) requires additional vigilance to manage potential difficulties in intubation. In our patient, craniofacial features, including micrognathia and macroglossia, created a potential for difficult intubation, consistent with these previous reports [4,8]. Thorough preparation and planning for such a scenario, with all available options, is essential. Although some authors have used a video laryngoscope and a smaller tube due to difficulty intubating with the conventional method [8] or employed a laryngeal mask for ventilation [10], in our case, standard Macintosh laryngoscopy was successful and effective. Nevertheless, alternative strategies were prepared, including supraglottic devices, videolaryngoscope, fiberoptic bronchoscope, and readiness for surgical airway. Preoperative evaluation in these patients is often limited. Caregivers play a critical role, not only in providing insight into the level of cooperation and potential anxiety triggers, but also in guiding the choice of sedation strategies and behavioral approaches [7].

Pharmacological premedication is a common approach for uncooperative patients with developmental disabilities, with benzodiazepine premedication considered routine in some centers [7], while oral midazolam and triazolam are effective in reducing preoperative anxiety [11]. In addition, inhalational induction with sevoflurane in a stress-free environment has been widely and successfully applied [12]. However, all pharmacological options were rejected by the patient, creating the need for a fully behavioral strategy. The caregiver-guided approach used - familiar verbal repetition and rhythmic music - is consistent with current evidence highlighting the importance of individualized, behaviorally oriented approaches in individuals with intellectual disabilities [13]. Parents and families should therefore be informed of feasible options regarding anesthetic induction and perioperative care, enabling them to actively contribute to the decision-making process. In our case, this strategy proved successful and underscores the value of including caregivers as active participants in perioperative planning.

Regarding the pharmacological agents used for anesthesia in these patients, the use of propofol and rocuronium for induction has been reported as safe in CDCS patients and was also well tolerated in our case. Anesthesia was maintained with sevoflurane. Although concerns have been raised regarding malignant hyperthermia-like reactions to sevoflurane in CDCS [14], inhaled sevoflurane is generally considered a safe option for maintenance of anesthesia [12,15]. In our patient, no adverse reactions occurred; nevertheless, vigilant intraoperative monitoring of temperature and end-tidal CO₂ was ensured throughout the procedure.

For analgesia, opioids have been used by other investigators [16,17], while in our case, opioid-free anesthesia was applied to enable smooth and rapid emergence. The combination of intravenous paracetamol and targeted local infiltration by the dental team provided adequate analgesia without the risk of respiratory depression. Avoiding opioids in patients with neurodevelopmental disorders can minimize postoperative agitation and facilitate same-day discharge. Fast-track recovery was a priority, given that prolonged hospital stays can increase agitation in patients with severe cognitive impairment. In our patient, discharge criteria were met promptly, with stable hemodynamics, effective pain control, and immediate reunion with his caregivers in the recovery area.

## Conclusions

This report demonstrates that in adult CDCS patients refusing premedication, individualized behavioral strategies can be central to achieving a smooth perioperative course. The participation of caregivers in the anesthetic process, meticulous airway preparation, and tailored opioid-free anesthesia together facilitated a safe, efficient, and complication-free experience. Particular attention is additionally required in the anesthetic management of such patients because of craniofacial dysmorphisms and the potential presence of associated comorbidities. Additional adult case reports are needed to strengthen evidence-based recommendations for this rare patient population, and future studies should aim to develop tailored anesthesia guidelines for adults with CDCS.
